# Emotional Dysregulation and Anxiety Control in the Psychopathological Mechanism Underlying Drive for Thinness

**DOI:** 10.3389/fpsyt.2014.00043

**Published:** 2014-04-24

**Authors:** Francesca Fiore, Giovanni M. Ruggiero, Sandra Sassaroli

**Affiliations:** ^1^Studi Cognitivi, Post-Graduate Cognitive Psychotherapy School, Milan, Italy; ^2^Psicoterapia Cognitiva e Ricerca, Post-Graduate Cognitive Psychotherapy School, Milan, Italy

**Keywords:** emotional dysregulation, control, mediation, therapy, drive for thinness

## Abstract

Emotional dysregulation is a process which consists in mitigating, intensifying, or maintaining a given emotion and is the trigger for some psychological disorders. Research has shown that an anxiety control plays an important role in emotional expression and regulation and, in addition, for anorexia nervosa (AN) and, more in general, in drive for thinness. Scientific literature suggests that in AN there is a core of emotional dysregulation and anxiety control. The aim of this study is to explore the roles of emotional dysregulation and anxiety control as independent or third variables in a mediational regression model related to drive for thinness. One hundred fifty-four clinical individuals with anorexia participated in the study and all completed a set of self-report questionnaires: eating disorders inventory version 3 (EDI-3), DERS, and the anxiety control questionnaire. The data confirmed a mediational model in which the relation between emotional dysregulation and drive for thinness is mediated by anxiety control. The current study partially supports a clinical model in which emotional dysregulation is a distal factor in eating disorders while the mediator variable anxiety control is a proximal factor in the psychopathological process underlying it.

## Introduction

Difficulties in modulating and regulating emotional arousal are a predisposing factor in emotional disorders, relational problems, and reduced well-being (1). The term emotional dysregulation refers to the many processes involved in mitigating, intensifying, and/or maintaining a given emotion. Regulatory processes can be automated or controlled, conscious or unconscious, but always involve long-lasting changes (2) and allow individuals to control their behavior in line with personal goals and environmental demands (3). The literature has long emphasized that cognitive control plays a role in the emergence of emotional disorders (4). In fact, attempts to control internal experiences such as unwanted thoughts or emotions can be factors in emotional disorders (5).

Scientific literature suggests that people use avoidant and/or impulsive affect regulation strategies (6). These have the dual effect of accentuating the intensity and frequency both of unwanted emotions and of negative mood. More specifically, these people show different difficulties in controlling their emotional experiences (7).

There is a growing consensus too about the role played by emotional dysregulation in eating disorders (8, 9). Emotion regulation problems were found across eating disorder subtypes. In a recent study, in which the emotional dysregulation is scored by Difficulties in Emotion Regulation Scale, it was obtained that patients with anorexia nervosa (AN) reported lower levels of emotional regulation (10, 11), and excessive exercising and dieting may serve as an excessive emotional regulation strategy (12). In support of these hypotheses, the literature identifies that in AN patients there is a core of emotional dysregulation (13), which, in turn, determines hyper-control. A high tendency to control and emotional dysregulation are, therefore, both constructs found in AN (14).

The assumption is that binging functions by negative reinforcement, that is by reducing or temporarily numbing negative emotions or distracting oneself from these aversive emotional states. This strategy can also be conceptualized as a controlling one (8). Control is an even more important cognitive aspect in AN, where patients’ sense of control is often obtained by continuously monitoring eating and body weight and shape (15). Dietary restrictions enhance the subjective perception of being in control (16, 17). Williams et al. (18) showed that individuals with AN perceive a low degree of internal control but high external control exerted by family and society. Serpell et al. (19) and Waller (20) have shown that gaining a sense of control and pride in the ability to control one’s own eating combats the feeling of being taken over by food thoughts or lacking control over personal thoughts about eating and weight (21, 22). The question is: how do they interact with each other?

### Aim of the study

The present study aimed to test if the process leading from either emotional dysregulation or control to the development of drive for thinness in individuals with AN or showing all criteria for AN except their weight falls within the normal range (corresponding to the diagnosis of eating disorder not otherwise specified; EDNOS) is mediational. In addition, the study aims to test if emotional dysregulation and control are independent or third variables in a mediation model. Dysregulation and control can be conceived as either proximal factors, a mediator playing the role of an on-the-spot cognitive state; or as distal factors, an independent initial variable related to long-term personality traits. A mediation model may thus provide important information about the psychopathological mechanism underlying drive for thinness in AN or in EDNOS.

## Materials and Methods

### Participants

Clinical participants were recruited from a population of 168 individuals requesting clinical help. The recruitment was carried out during the initial assessment phase in treatment for ED. We classified the participants as individuals with AN according to the Structured Clinical Interview axis I (SCID-I) for the IV-TR version of the Diagnostic and Statistical Manual of Mental Disorders (DSM). One hundred fifty-four Italian individuals with AN participated in the study (Table 1).

**Table 1:** **Descriptive statistics on the sample**.

Variables	*N* (%)
Male	21 (13.63)
Female	132 (85.71)
Mea age (SD) (*N* = 154)	36.85 (11.26)

The BMI of participants was 18.13 (SD 1.01). One hundred ten individuals had the full diagnosis of AN (71.4% of the sample) while 44 individuals had an EDNOS (28.6% of the sample) subtype diagnosis including all criteria for AN except their weight falls within the normal range.

We excluded from the study 14 individuals who had an ED other than AN, EDNOS including all criteria for AN except their weight falls within the normal range or had other ED diagnoses in addition to AN. All the clinical participants were treated as outpatients and received cognitive psychotherapy (one session per week).

The study was approved by the “Studi Cognitivi” and Milan Policlinico Ospedale Maggiore (Hospital) Institutional Review Board. All participants provided informed consent. The study participants received no compensation.

### Measures

Prior to taking part in the experiment, all subjects completed self-reports containing data regarding cognitive variables and ED criteria.

The self-report subscale drive for thinness from the Eating Disorders Inventory version 3 (EDI-3) was used to assess the dimension of AN or EDNOS including all criteria for AN except their weight falls within the normal range (23). Drive for thinness is a useful screening tool for ED. It is designed to tap into a core feature, namely excessive concern over dieting and fear of weight gain (24, 25). This subscale was devised on the basis of clinical conceptualizations by Bruch (26) and Russell (27). The use of individual subscales from a questionnaire is an accepted practice, if reliability indices are complied with. For example, Yoon et al. (28) used only one subscale from the Maslach Burnout Inventory and Archer and Thanzami (29) also used only one subscale from the Narcissistic Personality Inventory. In addition, Ruggiero et al. (30) and Sassaroli and Ruggiero (31) used the same one above-mentioned subscale from the EDI. In the current study, the Cronbach’s alpha values for these EDI subscales were acceptable, at over 0.7.

The Anxiety Control Questionnaire [ACQ; (32)] assesses perception of control over emotional reactions and external threats. It is designed to detect pathological perceptions of low control as well as an exaggerated fear of losing control. The ACQ is a 30-item questionnaire providing a total score that is the sum of scores on two subscales: the 16-item event subscale and the 14-item reactions subscale. Participants respond on a 6-point Likert Scale. Lower scores are attributed to individuals with an emotional disorder. We analyzed the ACQ total score because the psychometric properties of the composite score are stronger than those of the subscales taken individually (33). The total score has proven internally consistent, with high test–retest reliability, and is a valid basis for distinguishing between anxious and non-anxious individuals (32). Verification of the individual ACQ subscales would, therefore, have unnecessarily complicated the statistical analyses and weakened the tool’s psychometric qualities. Internal consistency measured by Cronbach’s α for each test administered was between 0.71 and 0.85 in this sample.

The Difficulties in Emotion Regulation Scale [DERS; (34)] was developed to assess emotion dysregulation more comprehensively than previously existing measures. The final dimension of DERS reflects an attempt to measure the flexible use of situationally appropriate strategies for modulating emotional responses. The DERS is a 36-item questionnaire providing a total score that is the sum of scores on six subscales: (a) lack of awareness of emotional responses, (b) lack of clarity of emotional responses, (c) non-acceptance of emotional responses, (d) limited access to emotion regulation strategies perceived as effective, (e) difficulties controlling impulses when experiencing negative emotions, and (f) difficulties engaging in goal-directed behaviors when experiencing negative emotions. Participants respond on a 5-point Likert Scale. Higher scores are attributed to individuals with a dysregulation disorder. We analyzed the DERS total score because the psychometric properties of the composite score are stronger than those of the subscales taken individually (34). The total score has proven internally consistent, with high test–retest reliability, and is a valid test for distinguishing the presence of dysregulation symptoms (35). Internal consistency measured by Cronbach’s α for each test administered was between 0.67 and 0.76 in this sample.

### Procedures

#### Mediation moderation analysis

The hypotheses were analyzed using a mediation regression analysis, with the goal of seeing the effect of the independent variable (emotional dysregulation) on the proposed mediator (control), and the effect of the proposed mediator on the dependent variable (drive for thinness). A bootstrapping method was used to assess for an indirect effect (36) with *n* = 5000 bootstrap re-sample. Bootstrapping is a non-parametric procedure that produces an approximation of the sample distribution of the indirect effects. This is achieved through empirically generating a sample (with replacement of size *n* = 5000) from the full data set and calculating the indirect effects in the re-sample. Bootstrapping confidence intervals and Sobel test (37) are usually chosen to test the indirect effect in the sampling distribution. Path estimates are calculated using OLS regression.

## Results

Given the few subjects with EDNOS on the sample, we decided to perform the analysis on all the samples. Pearson’s bivariate correlations regarding the ED group are included in Table 1. Notably, the drive for thinness was positively correlated with ACQ and DERS engagement, suggesting that ED patients report a substantial sense of control and emotional dysregulation (Table 2).

**Table 2:** **Pearson bivariate correlation, mean, standard deviation**.

Variables	1	2	3
DERS	–		
ACQ	−0.345**	–	
Drive for thinness	0.300**	0.243*	–
Mean	98.43	36.37	4.01
Standard deviation	22.61	14.52	5.83

*p < 0.05; **p < 0.01.

We would like to test a mediational model in which emotional dysregulation was considered the independent variable, while control was considered the mediator and drive for thinness a dependent variable. All of the tests confirmed that the distribution of residuals met the requirements for normality (Kolmogorov–Smirnov, Shapiro–Wilk, normal Q–Q plot, detrended normal Q–Q plot). Neither the histogram nor the probability–probability plot indicated that the assumption of residual normality was incorrect.

Prior to estimating the mediation model, effect models were calculated separately by a series of linear regression analysis performed to examine the association between DERS (predictor), ACQ (mediator), and drive for thinness (the criterion).

**Figure 1:**
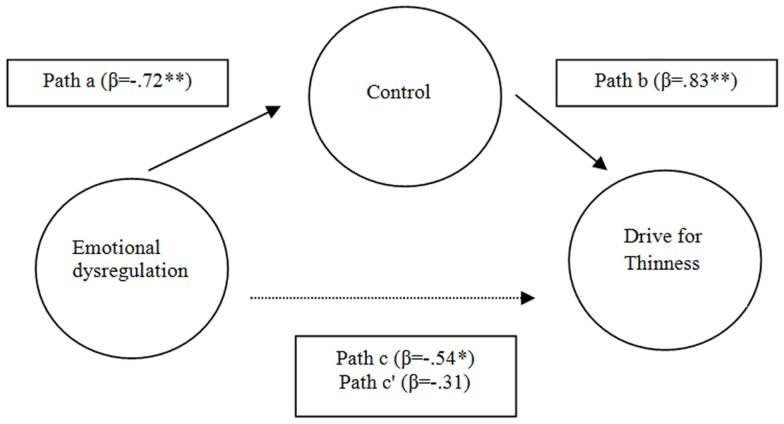
**The mediating role of control in relation between emotional dysregulation and drive for thinness**. **p < 0.01; *p < 0.05.

Step one: to test a Path c (Figure 1), we performed a linear regression analysis that showed DERS (the predictor) was significantly related to drive for thinness (the criterion) (β = 0.52, *p* = 0.00).
Step two: to investigate Path a (Figure 1), a regression analysis was conducted to examine the association between DERS (the predictor) and ACQ (the proposed mediator). The results indicate that DERS was significantly associated with ACQ (β = −0.72, *p* < 0.001).
Step three: a regression analysis was performed to observe Path b (Figure 1) in the model, or the association between ACQ (the proposed mediator) and drive for thinness (the criterion). The findings revealed that ACQ was significantly associated with DT (β = 0.83, *p* < 0.05).

Finally, a mediation test was conducted as recommended by Kenny et al. (35). In this case, the relation between DERS and DT was reduced and rendered non-significant (Path c = β = 0.31 versus path c = β = −0.54) by the inclusion of ACQ into the model, indicating a presence of mediation. Both bootstrapping and Sobel tests were used to confirm the mediation findings. Indeed, the bootstrapped 95% confidence intervals with 5000 iterations were −0.88 to −0.08 (36), indicating mediation and the Sobel tests (36) also confirmed the reduction in the relation between DERS and DT when ACQ was introduced into the model (*Z* = −2.03, *p* < 0.05).

**Figure 2:**
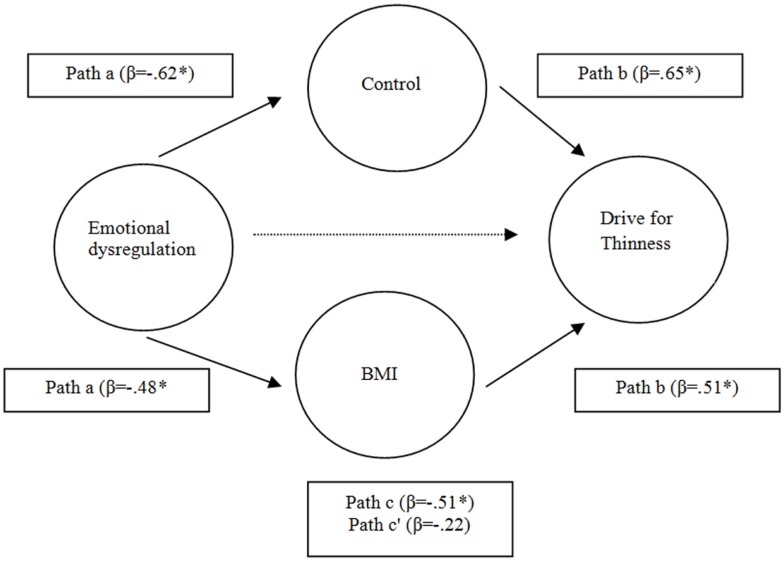
**The mediating role of control in relation and BMI between emotional dysregulation and drive for thinness**.*p < 0.05.

Thus, in order to explore the impact of BMI on these findings, we repeated the last analysis which included a BMI. The results indicated a reduction of the effect of the relationship between DERS, DT, and ACQ when was introduced into the model the BMI, but the model is confirmed to (*Z* = 1.56; *p* < 0.05) (Figure 2).

## Discussion

This study examined the role of emotional dysregulation and anxiety control in the psychopathological mechanism underlying drive for thinness and, consequently, AN or EDNOS including all criteria for AN except their weight falls within the normal range. We found that emotional dysregulation was significantly associated with drive for thinness. Specifically, this suggests that individuals who show increased emotional dysregulation report higher drive for thinness symptoms. We also found an association between emotional dysregulation and control. This finding is broadly consistent with Ruggiero and Sassaroli’s (30) work.

Finally, consistent with prediction, control was found to be a mediator of the association between emotional dysregulation and drive for thinness. Specifically, the inclusion of control in the model reduced the strength of the relation between emotional dysregulation and drive for thinness.

We can imagine that AN patients or EDNOS patients showing all criteria for AN except their weight falls within the normal range patients attempt to cope with the painful and terrific feelings associated with the lack of awareness and clarity of emotional responses and the difficulties in controlling negative emotions and impulses by using controlling strategies. The control is primarily behavioral, focused on strict dieting habits, and it is an attempt to surrogate the insufficient emotional regulation. This conception of control as a rigid and dysfunctional strategy of coping with dysregulated emotions may explain why control is a mediator variable that plays an intermediate role between emotional dysregulation and drive for thinness.

These results have clinically important implications. For example, when treating drive for thinness, interventions should take into account that the use of control in relation to emotional dysregulation produces restrictive behaviors. When treating drive for thinness, therapists can presume that control is the initial psychological attitude used by patients to cope with emotional dysregulation. In this case, they should encourage patients to see that their vulnerability is a consequence of their emotional dysregulation (13, 14).

The present study provides new insights into the roles of emotional dysregulation in AN or EDNOS including all criteria for AN except their weight falls within the normal range. Therefore, accounting for emotional dysregulation strategies seems not only important for treating drive for thinness in general but may further differentiate between subtypes in order to apply for disorder-specific difficulties. The specific role of emotion dysregulation in AN psychopathology has already been considered in adaptations of dialectical behavior therapy (13), and in specific treatment approaches for drive for thinness in AN or in EDNOS including all criteria for AN except their weight falls within the normal range such as emotion acceptance behavior therapy (38). However, further adaptations and differentiations of interventions that foster adaptive strategies in those domains of dysregulation that are specifically impaired, are desirable and suggested (39). Future research should resume investigating disorder-specific emotional dysregulation difficulties in order to enable the refinement of existing and the development of new treatment approaches for drive for thinness in ED.

Several limitations should be considered when interpreting the results of the present study. The first limitation was that we were not able to recruit only patients with a pure AN diagnosis but we had to add patients with EDNOS including all criteria for AN except their weight falls within the normal range.

In addition, the sample was extensively represented to female patients, limiting the generalizability to male ED patients. Although we examined larger samples than previous studies, larger samples would produce significant results, particularly in terms of difficulties of emotional awareness. Another limitation arises from the lack of systematically assessing lifetime diagnoses in the present study, which limits conclusions of emotional dysregulation specificity. Finally, some limitations also arise due to the exclusive administration of the DERS. Particularly, the DERS only measures one aspect of impulsivity (i.e., the self-evaluation of one’s capacity to remain in control of one’s behavior when experiencing negative emotions) and there also may be additional subtype-specific emotional dysregulation difficulties that were not assessed in the present study. Additionally, self-report measures are subject to memory biases and demand characteristics, and furthermore require rather high levels of introspection, which may not have been met by all participants. Future research should include more objective measures such as physiological variables and experimental designs in addition to self-report measures.

## Conflict of Interest Statement

The authors declare that the research was conducted in the absence of any commercial or financial relationships that could be construed as a potential conflict of interest.
